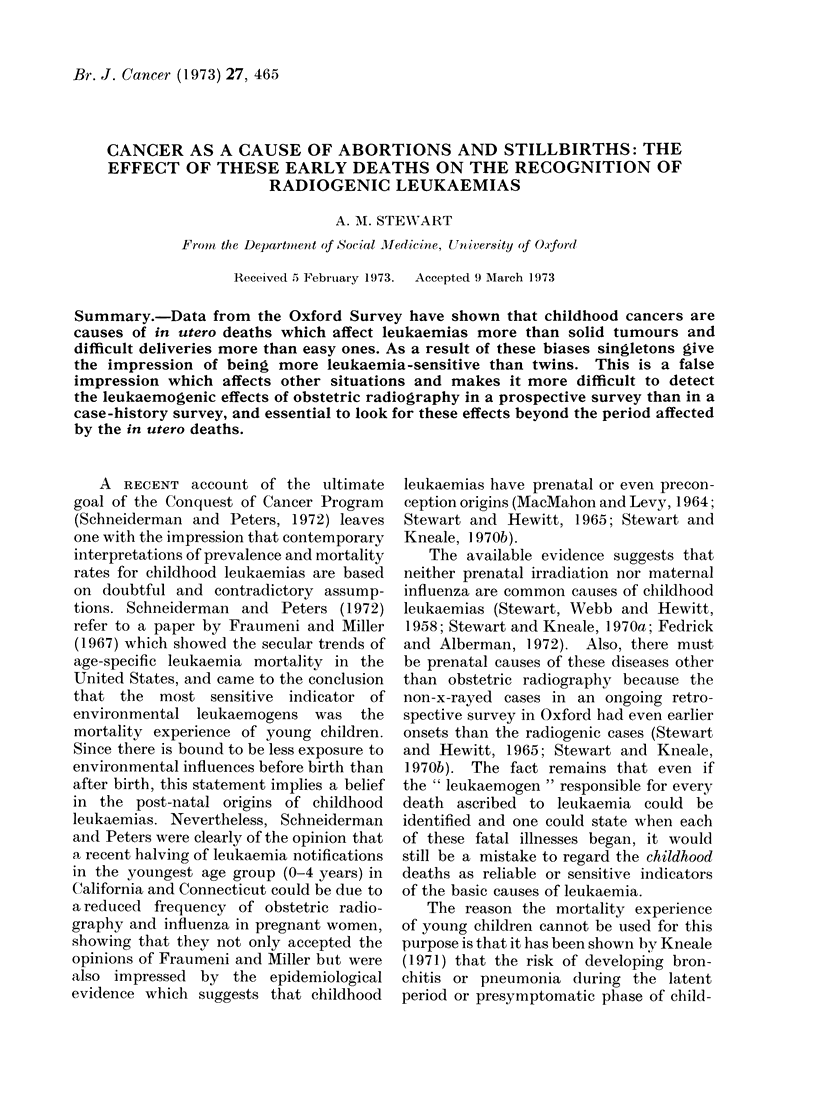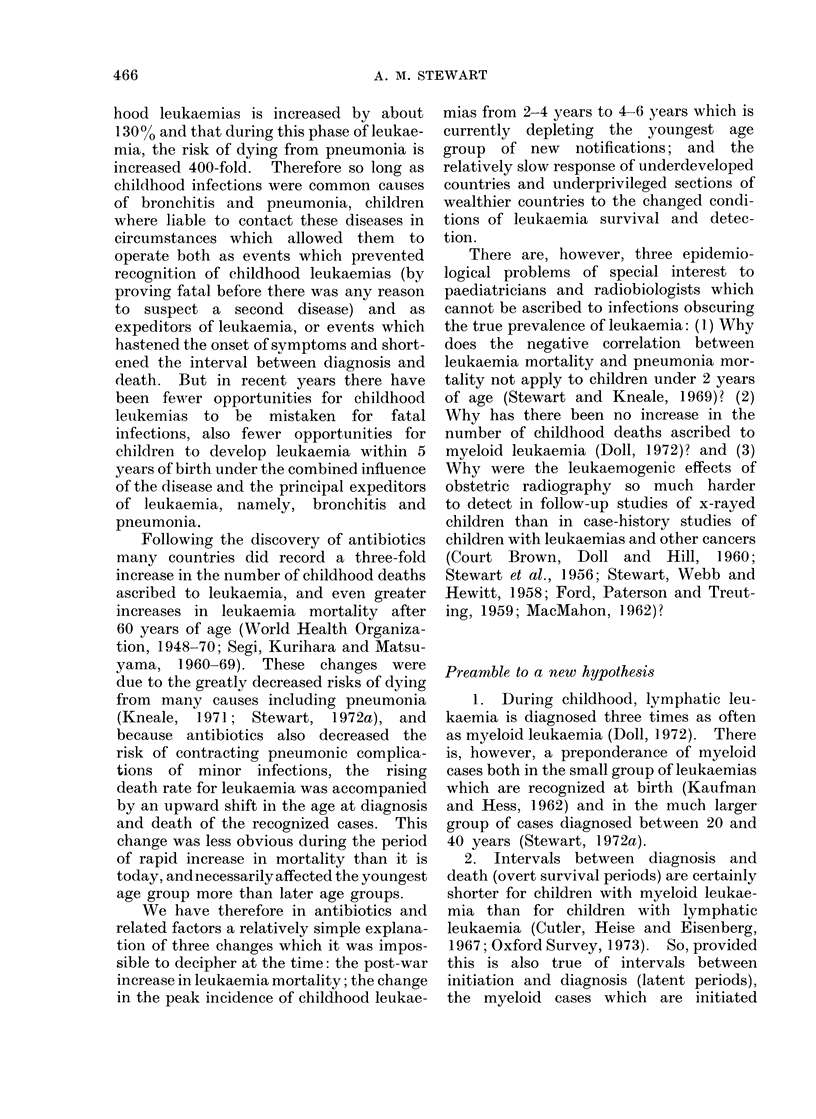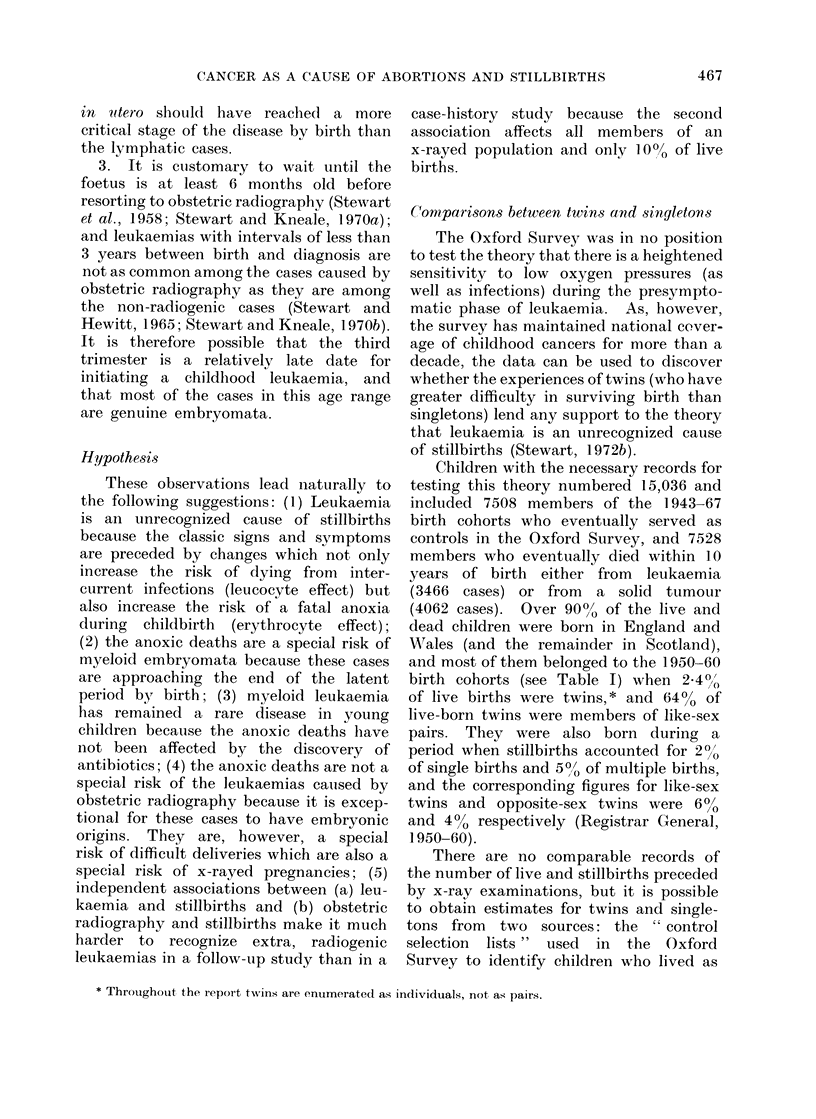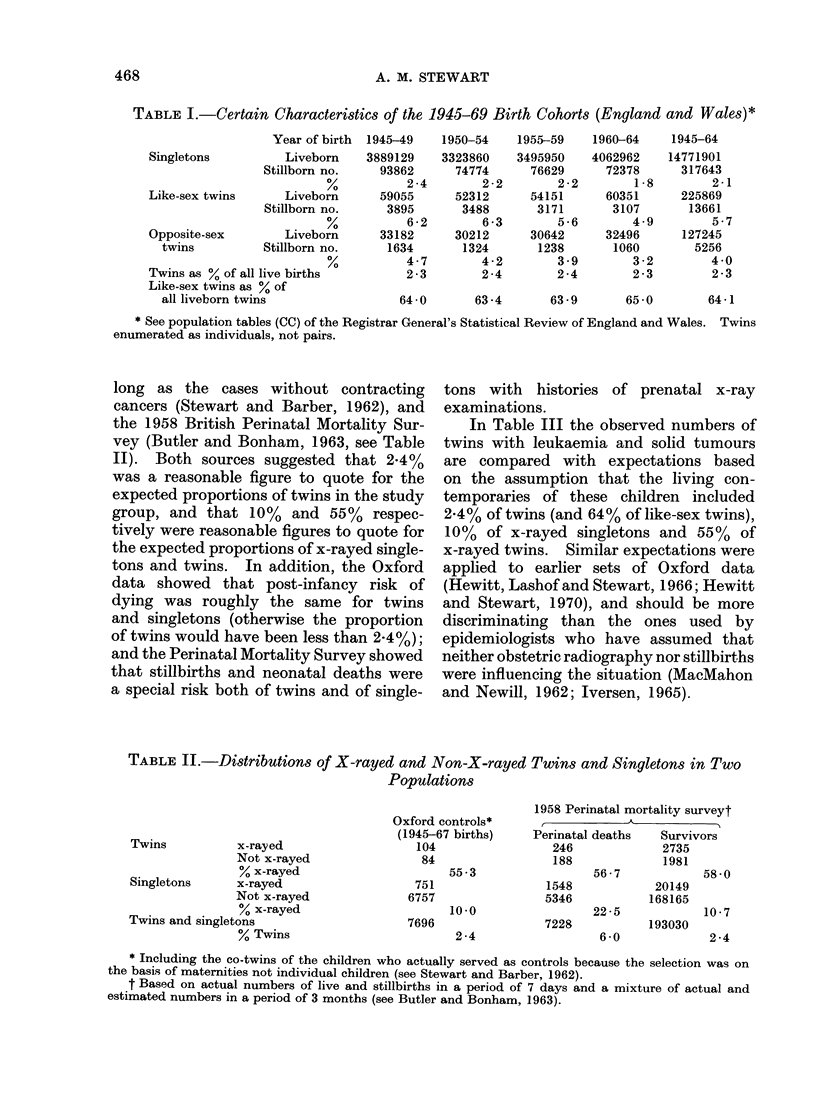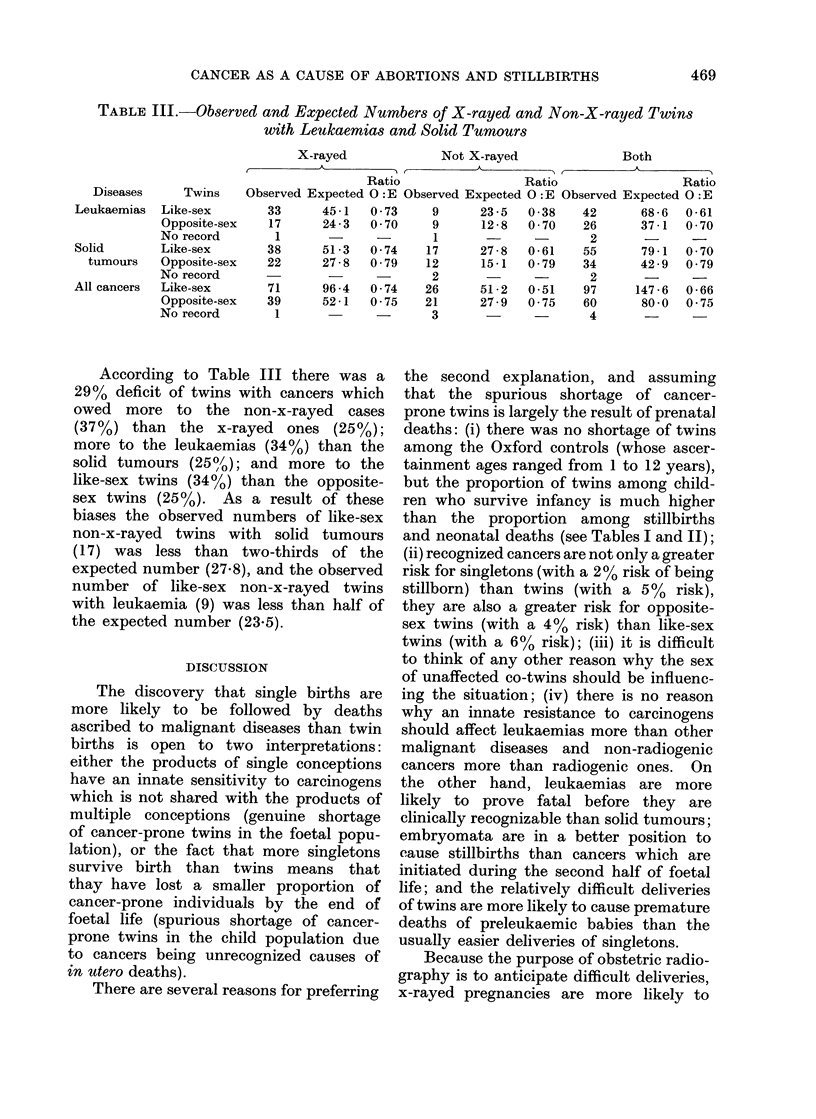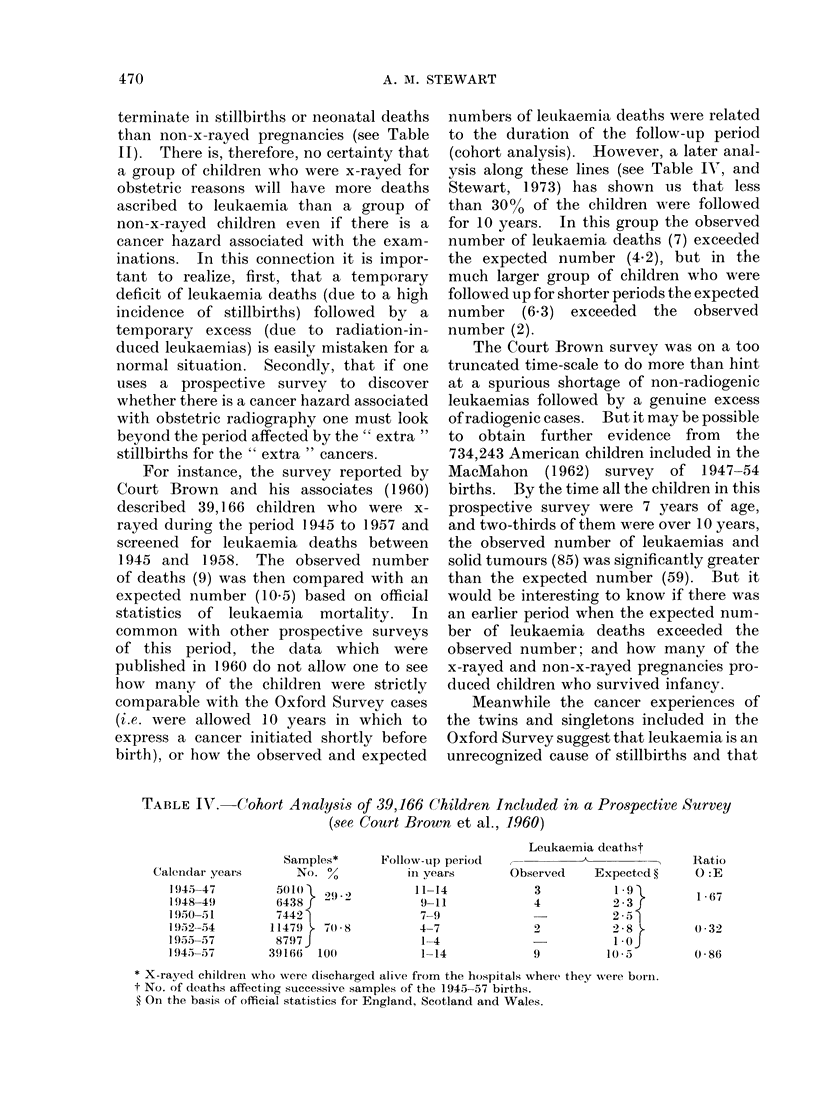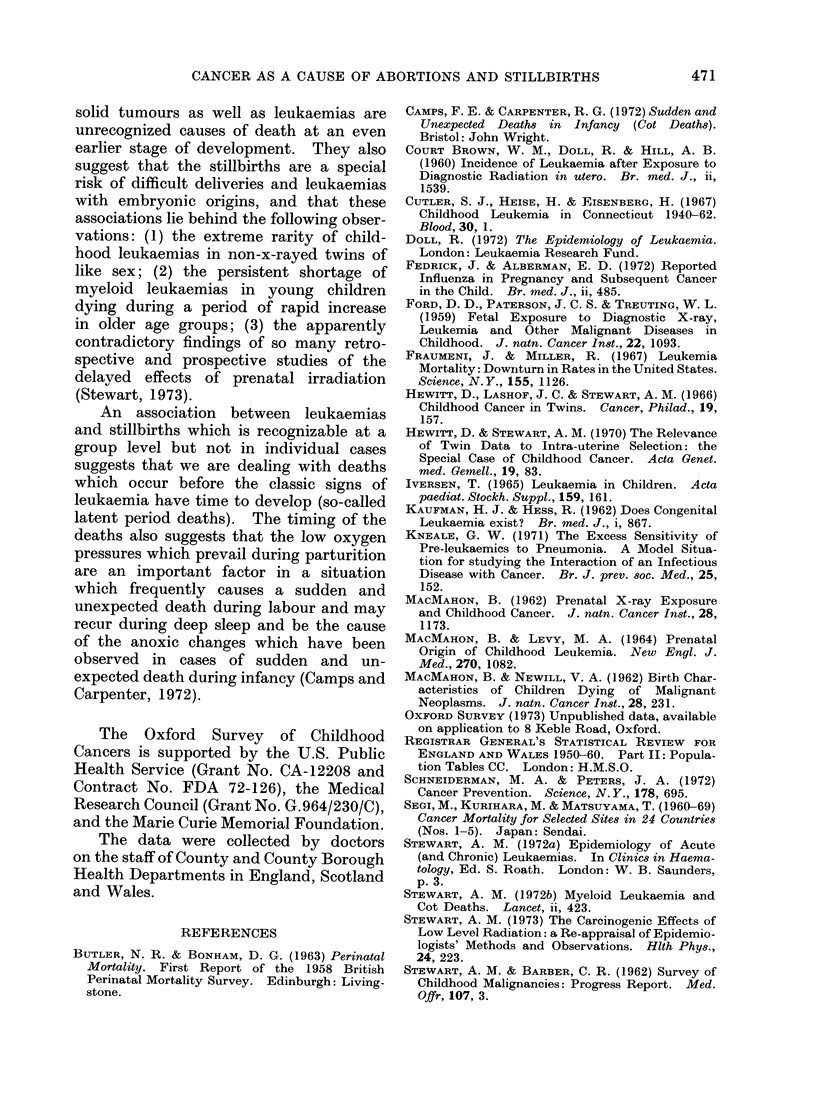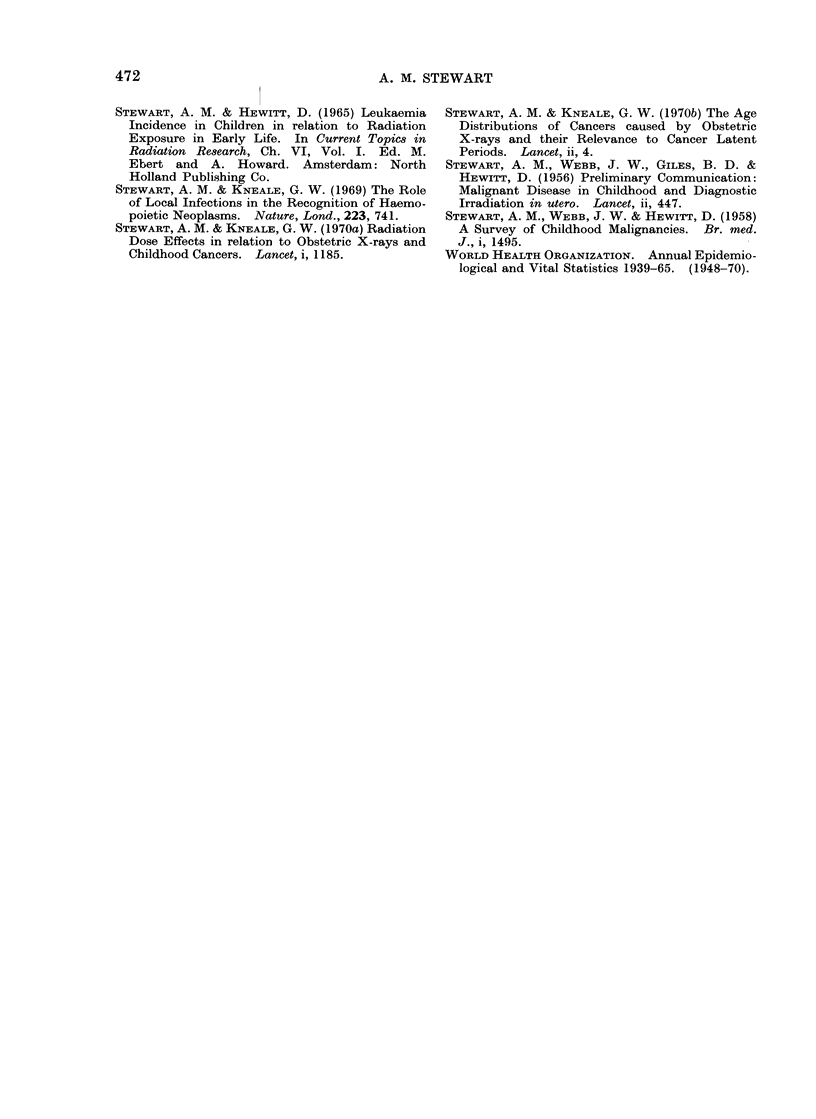# Cancer as a Cause of Abortions and Stillbirths: the Effect of these Early Deaths on the Recognition of Radiogenic Leukaemias

**DOI:** 10.1038/bjc.1973.59

**Published:** 1973-06

**Authors:** A. M. Stewart

## Abstract

Data from the Oxford Survey have shown that childhood cancers are causes of *in utero* deaths which affect leukaemias more than solid tumours and difficult deliveries more than easy ones. As a result of these biases singletons give the impression of being more leukaemia-sensitive than twins. This is a false impression which affects other situations and makes it more difficult to detect the leukaemogenic effects of obstetric radiography in a prospective survey than in a case-history survey, and essential to look for these effects beyond the period affected by the *in utero* deaths.


*Br. J. Cancer* (1973) **27**, 465

### CANCER AS A CAUSE OF ABORTIONS AND STILLBIRTHS: THE EFFECT OF THESE EARLY DEATHS ON THE RECOGNITION OF RADIOGENIC LEUKAEMIAS

A. M. STEWART

*From the Department of Social Medicine, University of Oxford*

Received 5 February 1973. Accepted 9 March 1973

**Summary.—Data from the Oxford Survey have shown that childhood cancers are causes of *in utero* deaths which affect leukaemias more than solid tumours and difficult deliveries more than easy ones. As a result of these biases singletons give the impression of being more leukaemia-sensitive than twins. This is a false impression which affects other situations and makes it more difficult to detect the leukaemogenic effects of obstetric radiography in a prospective survey than in a case-history survey, and essential to look for these effects beyond the period affected by the *in utero* deaths.**

A RECENT account of the ultimate goal of the Conquest of Cancer Program (Schneiderman and Peters, 1972) leaves one with the impression that contemporary interpretations of prevalence and mortality rates for childhood leukaemias are based on doubtful and contradictory assumptions. Schneiderman and Peters (1972) refer to a paper by Fraumeni and Miller (1967) which showed the secular trends of age-specific leukaemia mortality in the United States, and came to the conclusion that the most sensitive indicator of environmental leukaemogens was the mortality experience of young children. Since there is bound to be less exposure to environmental influences before birth than after birth, this statement implies a belief in the post-natal origins of childhood leukaemias. Nevertheless, Schneiderman and Peters were clearly of the opinion that a recent halving of leukaemia notifications in the youngest age group (0–4 years) in California and Connecticut could be due to a reduced frequency of obstetric radiography and influenza in pregnant women, showing that they not only accepted the opinions of Fraumeni and Miller but were also impressed by the epidemiological evidence which suggests that childhood leukaemias have prenatal or even preconception origins (MacMahon and Levy, 1964; Stewart and Hewitt, 1965; Stewart and Kneale, 1970*b*).

The available evidence suggests that neither prenatal irradiation nor maternal influenza are common causes of childhood leukaemias (Stewart, Webb and Hewitt, 1958; Stewart and Kneale, 1970*a*; Fedrick and Alberman, 1972). Also, there must be prenatal causes of these diseases other than obstetric radiography because the non-x-rayed cases in an ongoing retrospective survey in Oxford had even earlier onsets than the radiogenic cases (Stewart and Hewitt, 1965; Stewart and Kneale, 1970*b*). The fact remains that even if the " leukaemogen " responsible for every death ascribed to leukaemia could be identified and one could state when each of these fatal illnesses began, it would still be a mistake to regard the *childhood* deaths as reliable or sensitive indicators of the basic causes of leukaemia.

The reason the mortality experience of young children cannot be used for this purpose is that it has been shown by Kneale (1971) that the risk of developing bronchitis or pneumonia during the latent period or presymptomatic phase of childhood leukaemias is increased by about 130% and that during this phase of leukaemia, the risk of dying from pneumonia is increased 400-fold. Therefore so long as childhood infections were common causes of bronchitis and pneumonia, children where liable to contact these diseases in circumstances which allowed them to operate both as events which prevented recognition of childhood leukaemias (by proving fatal before there was any reason to suspect a second disease) and as expeditors of leukaemia, or events which hastened the onset of symptoms and shortened the interval between diagnosis and death. But in recent years there have been fewer opportunities for childhood leukemias to be mistaken for fatal infections, also fewer opportunities for children to develop leukaemia within 5 years of birth under the combined influence of the disease and the principal expeditors of leukaemia, namely, bronchitis and pneumonia.

Following the discovery of antibiotics many countries did record a three-fold increase in the number of childhood deaths ascribed to leukaemia, and even greater increases in leukaemia mortality after 60 years of age (World Health Organization, 1948–70; Segi, Kurihara and Matsuyama, 1960–69). These changes were due to the greatly decreased risks of dying from many causes including pneumonia (Kneale, 1971; Stewart, 1972*a*), and because antibiotics also decreased the risk of contracting pneumonic complications of minor infections, the rising death rate for leukaemia was accompanied by an upward shift in the age at diagnosis and death of the recognized cases. This change was less obvious during the period of rapid increase in mortality than it is today, and necessarily affected the youngest age group more than later age groups.

We have therefore in antibiotics and related factors a relatively simple explanation of three changes which it was impossible to decipher at the time: the post-war increase in leukaemia mortality; the change in the peak incidence of childhood leukaemias from 2–4 years to 4–6 years which is currently depleting the youngest age group of new notifications; and the relatively slow response of underdeveloped countries and underprivileged sections of wealthier countries to the changed conditions of leukaemia survival and detection.

There are, however, three epidemiological problems of special interest to paediatricians and radiobiologists which cannot be ascribed to infections obscuring the true prevalence of leukaemia: (1) Why does the negative correlation between leukaemia mortality and pneumonia mortality not apply to children under 2 years of age (Stewart and Kneale, 1969)? (2) Why has there been no increase in the number of childhood deaths ascribed to myeloid leukaemia (Doll, 1972)? and (3) Why were the leukaemogenic effects of obstetric radiography so much harder to detect in follow-up studies of x-rayed children than in case-history studies of children with leukaemias and other cancers (Court Brown, Doll and Hill, 1960; Stewart *et al.*, 1956; Stewart, Webb and Hewitt, 1958; Ford, Paterson and Treuting, 1959; MacMahon, 1962)?

*Preamble to a new hypothesis*

1. During childhood, lymphatic leukaemia is diagnosed three times as often as myeloid leukaemia (Doll, 1972). There is, however, a preponderance of myeloid cases both in the small group of leukaemias which are recognized at birth (Kaufman and Hess, 1962) and in the much larger group of cases diagnosed between 20 and 40 years (Stewart, 1972*a*).

2. Intervals between diagnosis and death (overt survival periods) are certainly shorter for children with myeloid leukaemia than for children with lymphatic leukaemia (Cutler, Heise and Eisenberg, 1967; Oxford Survey, 1973). So, provided this is also true of intervals between initiation and diagnosis (latent periods), the myeloid cases which are initiated

*in utero* should have reached a more critical stage of the disease by birth than the lymphatic cases.

3. It is customary to wait until the foetus is at least 6 months old before resorting to obstetric radiography (Stewart *et al.*, 1958; Stewart and Kneale, 1970*a*); and leukaemias with intervals of less than 3 years between birth and diagnosis are not as common among the cases caused by obstetric radiography as they are among the non-radiogenic cases (Stewart and Hewitt, 1965; Stewart and Kneale, 1970*b*). It is therefore possible that the third trimester is a relatively late date for initiating a childhood leukaemia, and that most of the cases in this age range are genuine embryomata.

### *Hypothesis*

These observations lead naturally to the following suggestions: (1) Leukaemia is an unrecognized cause of stillbirths because the classic signs and symptoms are preceded by changes which not only increase the risk of dying from intercurrent infections (leucocyte effect) but also increase the risk of a fatal anoxia during childbirth (erythrocyte effect); (2) the anoxic deaths are a special risk of myeloid embryomata because these cases are approaching the end of the latent period by birth; (3) myeloid leukaemia has remained a rare disease in young children because the anoxic deaths have not been affected by the discovery of antibiotics; (4) the anoxic deaths are not a special risk of the leukaemias caused by obstetric radiography because it is exceptional for these cases to have embryonic origins. They are, however, a special risk of difficult deliveries which are also a special risk of x-rayed pregnancies; (5) independent associations between (a) leukaemia and stillbirths and (b) obstetric radiography and stillbirths make it much harder to recognize extra, radiogenic leukaemias in a follow-up study than in a case-history study because the second association affects all members of an x-rayed population and only 10% of live births.

### *Comparisons between twins and singletons*

The Oxford Survey was in no position to test the theory that there is a heightened sensitivity to low oxygen pressures (as well as infections) during the presymptomatic phase of leukaemia. As, however, the survey has maintained national coverage of childhood cancers for more than a decade, the data can be used to discover whether the experiences of twins (who have greater difficulty in surviving birth than singletons) lend any support to the theory that leukaemia is an unrecognized cause of stillbirths (Stewart, 1972*b*).

Children with the necessary records for testing this theory numbered 15,036 and included 7508 members of the 1943–67 birth cohorts who eventually served as controls in the Oxford Survey, and 7528 members who eventually died within 10 years of birth either from leukaemia (3466 cases) or from a solid tumour (4062 cases). Over 90% of the live and dead children were born in England and Wales (and the remainder in Scotland), and most of them belonged to the 1950–60 birth cohorts (see Table I) when 2·4% of live births were twins,* and 64% of live-born twins were members of like-sex pairs. They were also born during a period when stillbirths accounted for 2% of single births and 5% of multiple births, and the corresponding figures for like-sex twins and opposite-sex twins were 6% and 4% respectively (Registrar General, 1950–60).

There are no comparable records of the number of live and stillbirths preceded by x-ray examinations, but it is possible to obtain estimates for twins and singletons from two sources: the "control selection lists" used in the Oxford Survey to identify children who lived as

* Throughout the report twins are enumerated as individuals, not as pairs.

TABLE I.—*Certain Characteristics of the 1945–69 Birth Cohorts (England and Wales)**

| | Year of birth | 1945–49 | 1950–54 | 1955–59 | 1960–64 | 1945–64 |
|---|---|---|---|---|---|---|
| Singletons | Liveborn | 3889129 | 3323860 | 3495950 | 4062962 | 14771901 |
| | Stillborn no. | 93862 | 74774 | 76629 | 72378 | 317643 |
| | % | 2·4 | 2·2 | 2·2 | 1·8 | 2·1 |
| Like-sex twins | Liveborn | 59055 | 52312 | 54151 | 60351 | 225869 |
| | Stillborn no. | 3895 | 3488 | 3171 | 3107 | 13661 |
| | % | 6·2 | 6·3 | 5·6 | 4·9 | 5·7 |
| Opposite-sex twins | Liveborn | 33182 | 30212 | 30642 | 32496 | 127245 |
| | Stillborn no. | 1634 | 1324 | 1238 | 1060 | 5256 |
| | % | 4·7 | 4·2 | 3·9 | 3·2 | 4·0 |
| Twins as % of all live births | | 2·3 | 2·4 | 2·4 | 2·3 | 2·3 |
| Like-sex twins as % of all liveborn twins | | 64·0 | 63·4 | 63·9 | 65·0 | 64·1 |

* See population tables (CC) of the Registrar General's Statistical Review of England and Wales. Twins enumerated as individuals, not pairs.

long as the cases without contracting cancers (Stewart and Barber, 1962), and the 1958 British Perinatal Mortality Survey (Butler and Bonham, 1963, see Table II). Both sources suggested that 2·4% was a reasonable figure to quote for the expected proportions of twins in the study group, and that 10% and 55% respectively were reasonable figures to quote for the expected proportions of x-rayed singletons and twins. In addition, the Oxford data showed that post-infancy risk of dying was roughly the same for twins and singletons (otherwise the proportion of twins would have been less than 2·4%); and the Perinatal Mortality Survey showed that stillbirths and neonatal deaths were a special risk both of twins and of singletons with histories of prenatal x-ray examinations.

In Table III the observed numbers of twins with leukaemia and solid tumours are compared with expectations based on the assumption that the living contemporaries of these children included 2·4% of twins (and 64% of like-sex twins), 10% of x-rayed singletons and 55% of x-rayed twins. Similar expectations were applied to earlier sets of Oxford data (Hewitt, Lashof and Stewart, 1966; Hewitt and Stewart, 1970), and should be more discriminating than the ones used by epidemiologists who have assumed that neither obstetric radiography nor stillbirths were influencing the situation (MacMahon and Newill, 1962; Iversen, 1965).

TABLE II.—*Distributions of X-rayed and Non-X-rayed Twins and Singletons in Two Populations*

| | | Oxford controls* (1945–67 births) | 1958 Perinatal mortality survey† — Perinatal deaths | 1958 Perinatal mortality survey† — Survivors |
|---|---|---|---|---|
| Twins | x-rayed | 104 | 246 | 2735 |
| | Not x-rayed | 84 | 188 | 1981 |
| | % x-rayed | 55·3 | 56·7 | 58·0 |
| Singletons | x-rayed | 751 | 1548 | 20149 |
| | Not x-rayed | 6757 | 5346 | 168165 |
| | % x-rayed | 10·0 | 22·5 | 10·7 |
| Twins and singletons | | 7696 | 7228 | 193030 |
| | % Twins | 2·4 | 6·0 | 2·4 |

* Including the co-twins of the children who actually served as controls because the selection was on the basis of maternities not individual children (see Stewart and Barber, 1962).

† Based on actual numbers of live and stillbirths in a period of 7 days and a mixture of actual and estimated numbers in a period of 3 months (see Butler and Bonham, 1963).

TABLE III.—*Observed and Expected Numbers of X-rayed and Non-X-rayed Twins with Leukaemias and Solid Tumours*

| | | X-rayed | | | Not X-rayed | | | Both | | |
|---|---|---|---|---|---|---|---|---|---|---|
| Diseases | Twins | Observed | Expected | Ratio O:E | Observed | Expected | Ratio O:E | Observed | Expected | Ratio O:E |
| Leukaemias | Like-sex | 33 | 45·1 | 0·73 | 9 | 23·5 | 0·38 | 42 | 68·6 | 0·61 |
| | Opposite-sex | 17 | 24·3 | 0·70 | 9 | 12·8 | 0·70 | 26 | 37·1 | 0·70 |
| | No record | 1 | — | — | 1 | — | — | 2 | — | — |
| Solid tumours | Like-sex | 38 | 51·3 | 0·74 | 17 | 27·8 | 0·61 | 55 | 79·1 | 0·70 |
| | Opposite-sex | 22 | 27·8 | 0·79 | 12 | 15·1 | 0·79 | 34 | 42·9 | 0·79 |
| | No record | — | — | — | 2 | — | — | 2 | — | — |
| All cancers | Like-sex | 71 | 96·4 | 0·74 | 26 | 51·2 | 0·51 | 97 | 147·6 | 0·66 |
| | Opposite-sex | 39 | 52·1 | 0·75 | 21 | 27·9 | 0·75 | 60 | 80·0 | 0·75 |
| | No record | 1 | — | — | 3 | — | — | 4 | — | — |

According to Table III there was a 29% deficit of twins with cancers which owed more to the non-x-rayed cases (37%) than the x-rayed ones (25%); more to the leukaemias (34%) than the solid tumours (25%); and more to the like-sex twins (34%) than the opposite-sex twins (25%). As a result of these biases the observed numbers of like-sex non-x-rayed twins with solid tumours (17) was less than two-thirds of the expected number (27·8), and the observed number of like-sex non-x-rayed twins with leukaemia (9) was less than half of the expected number (23·5).

### DISCUSSION

The discovery that single births are more likely to be followed by deaths ascribed to malignant diseases than twin births is open to two interpretations: either the products of single conceptions have an innate sensitivity to carcinogens which is not shared with the products of multiple conceptions (genuine shortage of cancer-prone twins in the foetal population), or the fact that more singletons survive birth than twins means that thay have lost a smaller proportion of cancer-prone individuals by the end of foetal life (spurious shortage of cancer-prone twins in the child population due to cancers being unrecognized causes of *in utero* deaths).

There are several reasons for preferring the second explanation, and assuming that the spurious shortage of cancer-prone twins is largely the result of prenatal deaths: (i) there was no shortage of twins among the Oxford controls (whose ascertainment ages ranged from 1 to 12 years), but the proportion of twins among children who survive infancy is much higher than the proportion among stillbirths and neonatal deaths (see Tables I and II); (ii) recognized cancers are not only a greater risk for singletons (with a 2% risk of being stillborn) than twins (with a 5% risk), they are also a greater risk for opposite-sex twins (with a 4% risk) than like-sex twins (with a 6% risk); (iii) it is difficult to think of any other reason why the sex of unaffected co-twins should be influencing the situation; (iv) there is no reason why an innate resistance to carcinogens should affect leukaemias more than other malignant diseases and non-radiogenic cancers more than radiogenic ones. On the other hand, leukaemias are more likely to prove fatal before they are clinically recognizable than solid tumours; embryomata are in a better position to cause stillbirths than cancers which are initiated during the second half of foetal life; and the relatively difficult deliveries of twins are more likely to cause premature deaths of preleukaemic babies than the usually easier deliveries of singletons.

Because the purpose of obstetric radiography is to anticipate difficult deliveries, x-rayed pregnancies are more likely to terminate in stillbirths or neonatal deaths than non-x-rayed pregnancies (see Table II). There is, therefore, no certainty that a group of children who were x-rayed for obstetric reasons will have more deaths ascribed to leukaemia than a group of non-x-rayed children even if there is a cancer hazard associated with the examinations. In this connection it is important to realize, first, that a temporary deficit of leukaemia deaths (due to a high incidence of stillbirths) followed by a temporary excess (due to radiation-induced leukaemias) is easily mistaken for a normal situation. Secondly, that if one uses a prospective survey to discover whether there is a cancer hazard associated with obstetric radiography one must look beyond the period affected by the " extra " stillbirths for the " extra " cancers.

For instance, the survey reported by Court Brown and his associates (1960) described 39,166 children who were x-rayed during the period 1945 to 1957 and screened for leukaemia deaths between 1945 and 1958. The observed number of deaths (9) was then compared with an expected number (10·5) based on official statistics of leukaemia mortality. In common with other prospective surveys of this period, the data which were published in 1960 do not allow one to see how many of the children were strictly comparable with the Oxford Survey cases (*i.e.* were allowed 10 years in which to express a cancer initiated shortly before birth), or how the observed and expected numbers of leukaemia deaths were related to the duration of the follow-up period (cohort analysis). However, a later analysis along these lines (see Table IV, and Stewart, 1973) has shown us that less than 30% of the children were followed for 10 years. In this group the observed number of leukaemia deaths (7) exceeded the expected number (4·2), but in the much larger group of children who were followed up for shorter periods the expected number (6·3) exceeded the observed number (2).

The Court Brown survey was on a too truncated time-scale to do more than hint at a spurious shortage of non-radiogenic leukaemias followed by a genuine excess of radiogenic cases. But it may be possible to obtain further evidence from the 734,243 American children included in the MacMahon (1962) survey of 1947–54 births. By the time all the children in this prospective survey were 7 years of age, and two-thirds of them were over 10 years, the observed number of leukaemias and solid tumours (85) was significantly greater than the expected number (59). But it would be interesting to know if there was an earlier period when the expected number of leukaemia deaths exceeded the observed number; and how many of the x-rayed and non-x-rayed pregnancies produced children who survived infancy.

Meanwhile the cancer experiences of the twins and singletons included in the Oxford Survey suggest that leukaemia is an unrecognized cause of stillbirths and that

TABLE IV.—*Cohort Analysis of 39,166 Children Included in a Prospective Survey (see Court Brown* et al., *1960)*

| Calendar years | Samples* No. | % | Follow-up period in years | Leukaemia deaths† Observed | Expected § | Ratio O:E |
|---|---|---|---|---|---|---|
| 1945–47 | 5010 | 29·2 | 11–14 | 3 | 1·9 | 1·67 |
| 1948–49 | 6438 | | 9–11 | 4 | 2·3 | |
| 1950–51 | 7442 | 70·8 | 7–9 | — | 2·5 | 0·32 |
| 1952–54 | 11479 | | 4–7 | 2 | 2·8 | |
| 1955–57 | 8797 | | 1–4 | — | 1·0 | |
| 1945–57 | 39166 | 100 | 1–14 | 9 | 10·5 | 0·86 |

* X-rayed children who were discharged alive from the hospitals where they were born.
† No. of deaths affecting successive samples of the 1945–57 births.
§ On the basis of official statistics for England, Scotland and Wales.

solid tumours as well as leukaemias are unrecognized causes of death at an even earlier stage of development. They also suggest that the stillbirths are a special risk of difficult deliveries and leukaemias with embryonic origins, and that these associations lie behind the following observations: (1) the extreme rarity of childhood leukaemias in non-x-rayed twins of like sex; (2) the persistent shortage of myeloid leukaemias in young children dying during a period of rapid increase in older age groups; (3) the apparently contradictory findings of so many retrospective and prospective studies of the delayed effects of prenatal irradiation (Stewart, 1973).

An association between leukaemias and stillbirths which is recognizable at a group level but not in individual cases suggests that we are dealing with deaths which occur before the classic signs of leukaemia have time to develop (so-called latent period deaths). The timing of the deaths also suggests that the low oxygen pressures which prevail during parturition are an important factor in a situation which frequently causes a sudden and unexpected death during labour and may recur during deep sleep and be the cause of the anoxic changes which have been observed in cases of sudden and unexpected death during infancy (Camps and Carpenter, 1972).

The Oxford Survey of Childhood Cancers is supported by the U.S. Public Health Service (Grant No. CA-12208 and Contract No. FDA 72-126), the Medical Research Council (Grant No. G.964/230/C), and the Marie Curie Memorial Foundation.

The data were collected by doctors on the staff of County and County Borough Health Departments in England, Scotland and Wales.

### REFERENCES

BUTLER, N. R. & BONHAM, D. G. (1963) *Perinatal Mortality*. First Report of the 1958 British Perinatal Mortality Survey. Edinburgh: Livingstone.

CAMPS, F. E. & CARPENTER, R. G. (1972) *Sudden and Unexpected Deaths in Infancy (Cot Deaths)*. Bristol: John Wright.

COURT BROWN, W. M., DOLL, R. & HILL, A. B. (1960) Incidence of Leukaemia after Exposure to Diagnostic Radiation *in utero*. *Br. med. J.*, ii, 1539.

CUTLER, S. J., HEISE, H. & EISENBERG, H. (1967) Childhood Leukemia in Connecticut 1940–62. *Blood*, **30**, 1.

DOLL, R. (1972) *The Epidemiology of Leukaemia*. London: Leukaemia Research Fund.

FEDRICK, J. & ALBERMAN, E. D. (1972) Reported Influenza in Pregnancy and Subsequent Cancer in the Child. *Br. med. J.*, ii, 485.

FORD, D. D., PATERSON, J. C. S. & TREUTING, W. L. (1959) Fetal Exposure to Diagnostic X-ray, Leukemia and Other Malignant Diseases in Childhood. *J. natn. Cancer Inst.*, **22**, 1093.

FRAUMENI, J. & MILLER, R. (1967) Leukemia Mortality: Downturn in Rates in the United States. *Science, N.Y.*, **155**, 1126.

HEWITT, D., LASHOF, J. C. & STEWART, A. M. (1966) Childhood Cancer in Twins. *Cancer, Philad.*, **19**, 157.

HEWITT, D. & STEWART, A. M. (1970) The Relevance of Twin Data to Intra-uterine Selection: the Special Case of Childhood Cancer. *Acta Genet. med. Gemell.*, **19**, 83.

IVERSEN, T. (1965) Leukaemia in Children. *Acta paediat. Stockh. Suppl.*, **159**, 161.

KAUFMAN, H. J. & HESS, R. (1962) Does Congenital Leukaemia exist? *Br. med. J.*, i, 867.

KNEALE, G. W. (1971) The Excess Sensitivity of Pre-leukaemics to Pneumonia. A Model Situation for studying the Interaction of an Infectious Disease with Cancer. *Br. J. prev. soc. Med.*, **25**, 152.

MACMAHON, B. (1962) Prenatal X-ray Exposure and Childhood Cancer. *J. natn. Cancer Inst.*, **28**, 1173.

MACMAHON, B. & LEVY, M. A. (1964) Prenatal Origin of Childhood Leukemia. *New Engl. J. Med.*, **270**, 1082.

MACMAHON, B. & NEWILL, V. A. (1962) Birth Characteristics of Children Dying of Malignant Neoplasms. *J. natn. Cancer Inst.*, **28**, 231.

OXFORD SURVEY (1973) Unpublished data, available on application to 8 Keble Road, Oxford.

REGISTRAR GENERAL'S STATISTICAL REVIEW FOR ENGLAND AND WALES 1950–60. Part II: Population Tables CC. London: H.M.S.O.

SCHNEIDERMAN, M. A. & PETERS, J. A. (1972) Cancer Prevention. *Science, N.Y.*, **178**, 695.

SEGI, M., KURIHARA, M. & MATSUYAMA, T. (1960–69) *Cancer Mortality for Selected Sites in 24 Countries* (Nos. 1–5). Japan: Sendai.

STEWART, A. M. (1972*a*) Epidemiology of Acute (and Chronic) Leukaemias. In *Clinics in Haematology*, Ed. S. Roath. London: W. B. Saunders, p. 3.

STEWART, A. M. (1972*b*) Myeloid Leukaemia and Cot Deaths. *Lancet*, ii, 423.

STEWART, A. M. (1973) The Carcinogenic Effects of Low Level Radiation: a Re-appraisal of Epidemiologists' Methods and Observations. *Hlth Phys.*, **24**, 223.

STEWART, A. M. & BARBER, C. R. (1962) Survey of Childhood Malignancies: Progress Report. *Med. Offr*, **107**, 3.

Stewart, A. M. & Hewitt, D. (1965) Leukaemia Incidence in Children in relation to Radiation Exposure in Early Life. In *Current Topics in Radiation Research*, Ch. VI, Vol. I. Ed. M. Ebert and A. Howard. Amsterdam: North Holland Publishing Co.

Stewart, A. M. & Kneale, G. W. (1969) The Role of Local Infections in the Recognition of Haemopoietic Neoplasms. *Nature, Lond.*, **223**, 741.

Stewart, A. M. & Kneale, G. W. (1970*a*) Radiation Dose Effects in relation to Obstetric X-rays and Childhood Cancers. *Lancet*, i, 1185.

Stewart, A. M. & Kneale, G. W. (1970*b*) The Age Distributions of Cancers caused by Obstetric X-rays and their Relevance to Cancer Latent Periods. *Lancet*, ii, 4.

Stewart, A. M., Webb, J. W., Giles, B. D. & Hewitt, D. (1956) Preliminary Communication: Malignant Disease in Childhood and Diagnostic Irradiation *in utero*. *Lancet*, ii, 447.

Stewart, A. M., Webb, J. W. & Hewitt, D. (1958) A Survey of Childhood Malignancies. *Br. med. J.*, i, 1495.

World Health Organization. Annual Epidemiological and Vital Statistics 1939–65. (1948–70).